# hUCMSCs reduce theca interstitial cells apoptosis and restore ovarian function in premature ovarian insufficiency rats through regulating NR4A1-mediated mitochondrial mechanisms

**DOI:** 10.1186/s12958-022-00992-5

**Published:** 2022-08-19

**Authors:** Qianqian Luo, Yu Tang, Zhonglin Jiang, Hongchu Bao, Qiang Fu, Hongqin Zhang

**Affiliations:** 1 Xu Rongxiang Regenerative Medicine Research Center, Binzhou Medical University, Yantai, 264003 Shandong China; 2 Basic Medical College, Binzhou Medical University, Yantai, 264003 China; 3 Department of Clinical Medicine, Yantai Yuhuangding Hospital, Yantai, 264000 China; 4 School of Pharmacology, Institute of Aging Medicine, Binzhou Medical University, Yantai, 264003 China

**Keywords:** Premature ovarian insufficiency, hUCMSCs, Theca interstitial cells, NR4A1, Mitochondria

## Abstract

**Background:**

Human umbilical cord mesenchymal stem cells (hUCMSCs, retrospectively registered) have a lot of promise for treating theca interstitial cells(TICs) dysfunction in premature ovarian insufficiency (POI). The mechanisms, however, are still unknown.

**Methods:**

To examine the therapeutic and find the cause, we used both in vivo cisplatin-induced POI rat model and in vitro TICs model. HUCMSCs were injected into the tail veins of POI rats in an in vivo investigation. Then, using ELISA, HE staining, TUNEL apoptosis test kit, immunohistochemistry and western blot, researchers examined hormonal levels, ovarian morphology, TICs apoptosis, NR4A1 and Cyp17a1 in response to cisplatin treatment and hUCMSCs. TICs were obtained from the ovaries of rats and treated with the cisplatin, hUCMSCs supernatant, and the antagonist of NR4A1——DIM-C-pPhOH. ELISA, immunofluorescence, flow cytometry, JC-1 labeling and western blot analysis were used to detect T levels, Cyp17a1, NR4A1, and the anti-apoptotic protein Bcl-2, as well as pro-apoptotic proteins Bax, caspase-9, caspase-3, and cytochrome C(cytc).

**Results:**

We discovered that hUCMSCs restored the ovarian function, particularly TICs function based on measures of Cyp17a1 and T expression. NR4A1 was found in ovarian TICs of each group and NR4A1 expression was lower in the POI rats but higher following hUCMSCs therapy. The apoptosis of TICs generated by cisplatin was reduced after treatment with hUCMSCs. In vitro, NR4A1 was expressed in the nucleus of TICs, and NR4A1 as well as phospho-NR4A1 were decreased, following the apoptosis of TICs was emerged after cisplatin treatment. Interestingly, the localization of NR4A1 was translocated from the nucleus to the cytoplasm due to cisplatin. HUCMSCs were able to boost NR4A1 and phospho-NR4A1 expression while TICs’ apoptosis and JC-1 polymorimonomor fluorescence ratios reduced. Furthermore, Bcl-2 expression dropped following cisplatin treatment, whereas Bax, cytc, caspase-9, and caspase-3 expression rose; however, hUCMSCs treatment reduced their expression. In addition, DIM-C-pPhOH had no effect on the NR4A1 expression, but it did increase the expression of apoptosis-related factors such as Bax, cytc, caspase-9, and caspase-3, causing the apoptosis of TICs.

**Conclusions:**

These data show that hUCMSCs therapy improves ovarian function in POI rats by inhibiting TICs apoptosis through regulating NR4A1 -mediated mitochondrial mechanisms.

**Supplementary Information:**

The online version contains supplementary material available at 10.1186/s12958-022-00992-5.

## Introduction

Cancer patients under the age of 40 are more likely to experience premature ovarian insufficiency (POI) as a result of chemotherapy or radiation [1, 2]. Because it induces apoptosis, cisplatin(CDDP), one of the most efficient chemotherapeutic drugs, is a substantial risk factor for POI [3–5]. Such anti-cancer treatments can further cause symptoms like amenorrhea, ovarian atrophy, sexual hypoactivity, and decreased fertility, as well as leading to a slew of other conditions like osteoporosis and cardiovascular disease [6, 7]. Previous research has found that POI is linked to granulosa cells damage and apoptosis [8], which can be aided by pro-apoptotic proteins released by theca interstitial cells (TICs) [9]. TICs are located in the inner layer of the follicular theca and have a width of 3–5 cells, represent a crucial component for follicular development and differentiation from stromal cells of the cortex [10, 11]. TICs also synthesize androgens to support the nutritional and structural needs of granulosa cells and oocytes, which are crucial for follicular development and ovulation [12]. However, due to the scarcity of information on POI mechanisms, the possible interaction between TICs and POI has yet to be established. It's possible that TICs-derived factors govern granulosa cell apoptosis during follicular atresia. Evidence suggests that the orphan nuclear receptor NR4A1 is expressed in TICs [13], and that NR4A1 regulates cell apoptosis in other organs via mitochondrial mechanisms [14]. As a result, we investigated the impact of TICs on POI and whether NR4A1-mediated mitochondrial pathways play a role in POI in our research.

This is an essential topic because there is now no viable treatment for POI due to a lack of understanding of its processes and complicated etiology [15]. MSCs transplantation significantly enhanced the number of ovarian follicles and estradiol concentrations in a POI mouse model according to a prior study [16, 17]. These data imply that MSCs may help to repair ovarian structure and function in POI. Two potential mechanisms for MSCs’ influence on POI have been hypothesized based on prior work [18–21]. The first is that oocytes and ovarian cells, in particular granulosa cells (GCs), were differentiated by MSCs, and the second is that MSCs were able to restore ovarian function through paracrine action. Uncertainty persists, nonetheless, surrounding the intricacies of how human umbilical cord mesenchymal stem cells (hUCMSCs) work to treat POI. Therefore, the aim of this work was to investigate the mechanisms and effects of hUCMSCs using in vivo within a cisplatin-induced POI rat model and in vitro within the theca interstitial cells (TICs) model. With the help of all these findings, several potential processes behind these impacts will be clarified.

## Methods

The Laboratory Animal Care and Use Guidelines, which describe accepted procedures, were followed in all of the investigations. Informed consent for the harvesting of umbilical cords was obtained from mothers who had delivered healthy newborns in hospitals. The Animal Care Committee of Binzhou Medical University gave its approval to the procedures for these animal tests. A statement identifying the institutional and/or licensing committee approval for the experiments is included as an added attachment to this report (No. 2022–178).

### Cultivation of hUCMSCs in vitro

Healthy newborns’ umbilical cords were used to collect human umbilical cord-derived mesenchymal stem cells(hUCMSCs). As previously mentioned [22, 23], hUCMSCs separation, growth, and identification were carried out. Cell morphology was examined under a light microscope while the P3 passages of hUCMSCs had reached roughly 70% confluency in order to confirm the phenotypic of hUCMSCs. Using flow cytometry, immunophenotypic traits were discovered. HLA-DR, CD34, CD45, CD73, CD90, and CD44 monoclonal antibodies were utilized. As negative controls, we employed mouse IgG2b and IgG1 kappa isotype controls (seen as PE and FITC). The manufacturers' specified concentrations for each factor were followed while using them. The creation of adipocytes and osteoblasts in accordance with the technique allowed researchers to identify the differentiation of hUCMSCs. Cells from the third to the fifth generation were chosen for these tests, and the hUCMSCs culture media from various batches was collected and stored separately for later research.

### The POI rat model and hUCMSCs treatment

Six-week-old female Wistar rats (*n* = 30) were provided by the Jinan Pengyue Laboratory Animal Breeding Co. LTD (Jinan, Shandong). All of the rats were kept in a colony room with the temperature (22 ± 2℃) and light cycle (lights on from 0700-1900 h). They were randomized into three groups at random: control, POI, and POI + hUCMSCs group (*n* = 10/group). For seven days, 2 mg/kg of cisplatin was administered intraperitoneally every day as part of the POI treatment [24]. The control group received an injection of saline in an identical volume. Rats within the POI + hUCMSCs group were injected with a hUCMSCs suspension via the tail vein consisting of 100 μl phosphate-buffered saline (PBS) containing 1 × 10^6^ hUCMSCs. Prior to injection, cell suspensions were well stirred. The injection site was squeezed using a medical tampon following treatment with hUCMSCs. After that, rats were separated into squirrel cages for monitoring. One week after treatment, ovaries and serum were removed at the proestrus stage for additional research. Daily vaginal smears were used to test the rat's estrus cycle. In addition, hUCMSCs were marked with GFP by lentivirus for 72 h (hanbio, shanghai, China, MOI = 10). Frozen sections were used to examine the location of hUCMSCs over 24 h, 72 h, 120 h, and 144 h. The nucleus was indicated by blue fluorescence while it was stained with DAPI.

### Isolation, culture, and identification of TICs

Ovaries from 4-week-old Wistar rats were also obtained using the methods outlined by Chen et al. [25]. To detach granulosa cell layers, the ovaries were placed in Leibovitz’s L-15 medium supplemented with 10% fetal bovine serum (FBS) (AusGeneX, Australia) and 1% penicillin and streptomycin. The remainder of each follicle was then separated from the TICs. Utilizing two surgical forceps for dissection and further dispersion, TICs were then treated with 0.5 mg/ml collagenase (GIBCO; USA), which was then dissolved with McCoy's 5A (Modified) Medium (GIBCO; USA) at 37 °C for 40 min. By shaking the cells for 20 min at a time, the cells were separated. TICs were suspended and grown in McCoy's 5A cultivated medium at 37 °C and 5% CO_2_ atmosphere within 6 well plastic plates after two rinses with McCoy's 5A cultivated medium, which contained McCoy's 5A media, 10% FBS (AusGeneX, Australia), and 1% penicillin and streptomycin (Corning; USA).

### Cisplatin treatments

TICs were planted in 6-well plates (1 105 cells/well) after achieving 80% confluence. For 22 h at 37 °C, cells were separated into three groups based on the various treatments: (1) the control group received untreated medium; (2) the CDDP group received CDDP (2 mg/L) alone; and (3) the CDDP + hUCMSCs group received CDDP + hUCMSCs supernatants. To ensure that the CDDP concentration was employed in vitro investigations, TICs were treated with various concentrations of CDDP (0–10 g/L) dissolved in the cultured medium. The volume ratio of hUCMSCs to TICs cultured media (Mc Coy's 5A medium containing 10% FBS) was 1:1 in the CDDP + hUCMSCs group. We used immunofluorescent, western blot, and flow cytometry tests to identify the expression of NR4A1, Cytochrome P450 17A1 (Cyp17a1), and TICs apoptosis, respectively.

### Enzyme-Linked Immunosorbent Assay (ELISA)

The rat ELISA kit’s instructions were followed to determine the amounts of follicle-stimulating hormone (FSH), luteinizing hormone (LH), estradiol (E_2_), and testosterone (T) levels in the serum as well as the levels of T and E_2_ in the culture medium for TICs.

### Hematoxylin and eosin staining

Each group's ovaries were taken out and fixed in 4% paraformaldehyde. Paraffin sects (5 μm thick) were prepared and stained with hematoxylin and eosin (HE), followed by observation under light microscopy. According to the quantification of follicular counts as stated in a prior work [21], the number of follicles within each developmental stage was counted. In order to avoid double counting of follicles, differential follicle counts were performed at consecutive intervals of 100 μm according to accepted definitions of follicular classification. When the nucleus could be seen clearly and was encircled by a single layer of flattened squamous follicular cells, a primordial follicle was considered to exist [26–28]. An oocyte that is encircled by a single layer of cuboidal granulosa cells is referred to as a primary follicle. A secondary follicle was only counted if the nucleus was visible and had two or more layers of cuboidal granulosa cells without an antrum.

### One step TUNEL apoptosis assay kit

According to the manufacturer's instructions, the one-step TUNEL apoptosis test kit (Elabscience, wuhan, China) was used to assess the apoptosis in the ovarian tissue slides. Each sample slice received 50 μl of TUNEL, which included enzyme solution (8 μl) and labeling solution (42 μl). Cells were stained with 4',6-diamidino-2-phenylindole(DAPI) to redden the nucleus. The ovaries were examined using laser confocal microscopy to look for TUNEL-positive cells.

### Immunohistochemistry or immunofluorescence

Sheep serum was used to block the paraffin ovary Sects. (5 μm) from each group as well as the TIC slides. The primary antibodies Cyp17a1 (1:100 dilution; ab125022; Abcam), FSHR (1:100 dilution; bs-20658R; Bioss), and NR4A1 (1:50 dilution; 12,235–1-AP; Proteintech) used in these reactions were incubated at 4℃ overnight with the second antibody (HRP or fluorescence) being added on the following day. The primary antibody was subjected to a negative control using PBS. Two pathologists separately examined each section under a microscope and conducted a semi-quantitative analysis.

### Western blot

Each group's fresh ovarian tissue was removed, and the P1 generation of TICs was cultivated until it reached 90% confluence. Nuclear protein extraction kit (Solarbio, Beijing, China)was used to extract nuclear and cytoplasmic protein from TICs as well as total protein using RIPA pyrolysis. SDS-PAGE was used to separate the proteins, and they were then moved to the polyvinylidene difluoride membrane (PVDF). The membranes were incubated with the primary antibodies Cyp17a1 (1:4000 dilution; ab125022; Abcam), NR4A1 (1:500 dilution; sc-166166; Santa Cruz), Phospho-NR4A1 (1:1000 dilution; Ser351; Cell signaling technology), Bcl-2 (1:1000 dilution; Abclonal), Bax (1:1000 dilution; Abclonal), Caspase-9(1:1000 dilution; Abclonal), caspase-3(1:500 dilution; ab32351; Abcam) and cytc(1:500 dilution; Cell signaling technology) at 4℃ overnight, following being washed three times with TBS added with Tween 20(TBST). The second antibody was administered at room temperature for one hour and was HRP-conjugated affinipure goat anti-rabbit IgG (H + L) (1:40,000 dilution; 10,285–1-AP; Proteintech). Utilizing the Tanon 5200 analytical equipment (Tanon, Shanghai, China), chemiluminescence was measured following reaction with the Ultra-sensitive chemiluminescence kit (Sparkjade, Qingdao, Shandong).

### JC-1 staining

With the aid of flow cytometry, the lipophilic cationic probe 5,5',6,6'-tetrachloro-1,1',3,3'-tetraethylbenzimidazole-carbocyanine iodide (JC-1, M8650, Solarbio) was identified as a direct correlate of mitochondrial membrane potential in living cells. For the TICs staining, the JC-1 solution was prepared in accordance with the kit's instructions. TICs were subjected to JC-1 solution for 20 min at 37 °C after being treated with cisplatin and hUCMSC. The mixtures with JC-1 solution were rapidly centrifuged at 600 g for 4 min, followed by two items of washing with JC-1 staining buffer (1 ×), and then resuspended in PBS. Red to green fluorescent ratios were determined using SPSS after the red-green fluorescence's intensity was assessed.

### Detection of TICs apoptosis

TICs were stained with Annexin V-FITC/PI to detect cell apoptosis, which was accomplished with the aid of flow cytometry. After being treated with cisplatin and hUCMSCs for 22 h at 37 °C, the TICs (10^5^ cells/100 μl) were centrifuged at 300 g to pellet them, and they were then resuspended in 1X binding buffer. After adding FITC Annexin V and PI (5 μl each), the cells were gently vortexed and incubated for 15 min at RT in the dark. After that, 400 μl of 1X binding buffer was added, and using a FACScan device, flow cytometry was employed for analysis. Cells considered as viable are FITC Annexin V and PI negative. FITC Annexin V positive and PI negative cells are in the early stages of apoptosis. Both FITC Annexin V and PI are positive in cells that are in late apoptosis or are already dead.

### Statistical analysis

All experimental data are shown as mean ± standard deviation (mean ± SD) and statistical differences were analyzed with the use of a one-way analysis of variance (ANOVA) and the Bonferroni test for post-hoc comparisons. A *P* < 0.05 was required for results to be considered statistically significant.

The results of the experiments are shown as mean ± standard deviation (mean ± SD) and statistical differences were examined using a one-way analysis of variance (ANOVA) and the Bonferroni test for post-hoc comparisons. Results were deemed statistically significant at *P* < 0.05.

## Results

### Identification of human umbilical cord mesenchymal stem cells (hUCMSCs) and homing to ovaries in rats

The P_3_ generation of hUCMSCs resembled fibroblast-like cells morphologically, and it took 48 h to double its number (Supplementary Fig. 1B). The phenotype for the third passage of hUCMSCs was determined utilizing flow cytometry, and the cell surface marker is depicted in Supplementary Fig. 1A. CD73, CD90, and CD44 were mesenchymal cell-specific markers that were expressed in hUCMSCs, whereas CD34, CD45, and HLA-DR were not. Furthermore, these hUCMSCs had multipotent effects since they could differentiate into osteoblasts and adipocytes (Supplementary Fig. 1C-D). These findings imply that the hUCMSCs obtained have characteristics similar to that of MSCs.

**Fig. 1:**
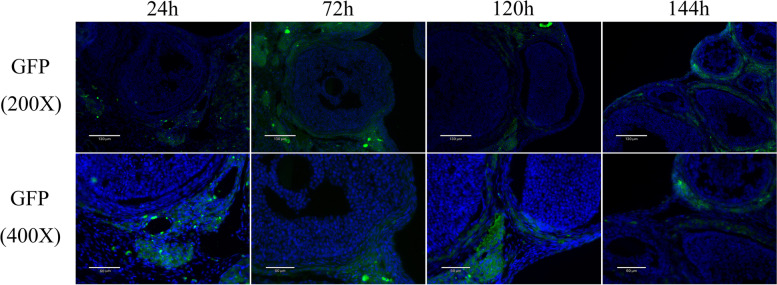
The location of hUCMSCs were observed over 24 h, 72 h, 120 h, and 144 h after GFP-labeled hUCMSCs transplantation. HUCMSCs were labeled by GFP(shown as green) and the nucleus was indicated by blue fluorescence while it was stained with DAPI. GFP: Green fluorescent protein, DAPI: 4’,6-diamidino-2-phenylindole, hUCMSCs: human umbilical cord mesenchymal stem cells. Scale bar: 130 μm and 60 μm

To further investigate whether hUCMSCs affects through direct or indirect effects, hUCMSCs were marked with GFP using lentivirus for 72 h. The pictures had been shown in supplementary Fig. 3. After GFP-labeled hUCMSCs transplantation, the location of hUCMSCs were observed over 24 h, 72 h, 120 h, and 144 h. The findings demonstrated that 24 h after hUCMSC transplantation, hUCMSCs were discovered in the ovarian interstitium; however, 72 h, 120 h, and 144 h later, hUCMSCs were discovered in the layer of TICs (Fig. 1). These findings also demonstrate the significance of TICs in the treatment of POI with hUCMSCs.

### Restoration of ovarian function in POI with hUCMSCs treatment -In vivo model

**Fig. 2:**
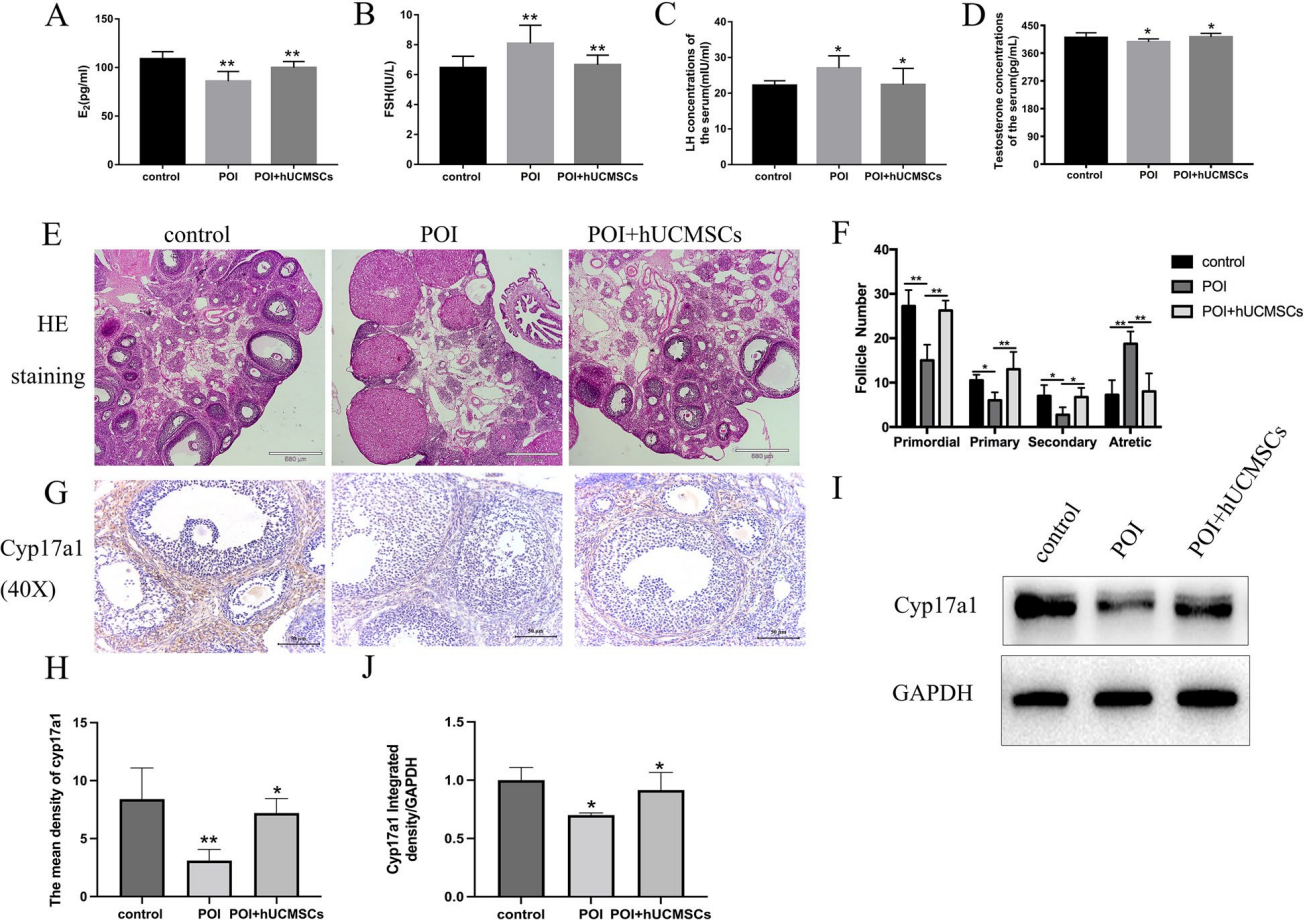
The analysis of follicular counts before and after treatment with hUCMSCs, as well as changes in morphology and the expression of Cyp17a1 in the ovaries. **A**-**D** ELISA was used to measure the levels of testosterone, FSH, LH, and E_2_, and SPSS was used to display the histograms of the results. **E**–**F** Changes in morphology and the quantity of follicles in the ovaries of the three groups. **G**-**J** Expression and the semi-quantitative analysis of Cyp17a1 within the ovary as determined with immunohistochemistry and western blot. FSH, Follicle-Stimulating Hormone; E_2_, estradiol. Scale bar = 580 μm and 50 μm. **:*P* < 0.01, *:*P* < 0.05

The cisplatin-induced rat model of POI was utilized to evaluate the impact of hUCMSCs on ovarian function in vivo. Changes in hormonal levels and ovarian morphology were taken as indications of ovarian function. FSH and LH levels in POI rats were considerably higher than those in the control group, but E_2_ levels fell. FSH, LH, and E_2_ levels were all returned to control levels when POI rats were treated with hUCMSCs (Fig. 2A-C). In this way, the hypoestrogenic and hyper-gonadotropin status resulting from POI was reinstated to that of normal levels following hUCMSCs treatment.

Additionally, the ovaries of these POI rats showed notable morphological alterations, including a decrease in the number of follicles and an increase in the amounts of luteinized components being observed in these rats. However, following the administration of hUCMSCs, significantly increases in the number of follicles were now present and mature follicles had re-emerged, as well as a few corpus luteum and atresia follicles. The number of follicles was indicated by these results. The number of primordial, primary, and secondary follicles dropped while atretic follicles rose in the POI group when compared to the control group. Primordial and developing follicles, including primary and secondary follicles, increased after hUCMSCs treatment while atretic follicles decreased (Fig. 2E-F). These results indicate that hUCMSCs may be able to restore ovarian function in this rat model of POI.

Moreover, the findings of the immunohistochemistry, western blot, and ELISA tests showed that Cyp17a1 and testosterone (T) expression were present, proving that the TICs function in these ovaries. Both Cyp17a1 expression in the ovary and T levels in the serum of POI rats were lower than those seen in the control group, but both increased following treatment with hUCMSCs (Fig. 2D, G-J). These data imply that POI was associated with TICs dysfunction, while hUCMSCs could ameliorate the symptom.

### The TICs’ apoptosis in POI with hUCMSCs treatment -In vivo model

**Fig. 3:**
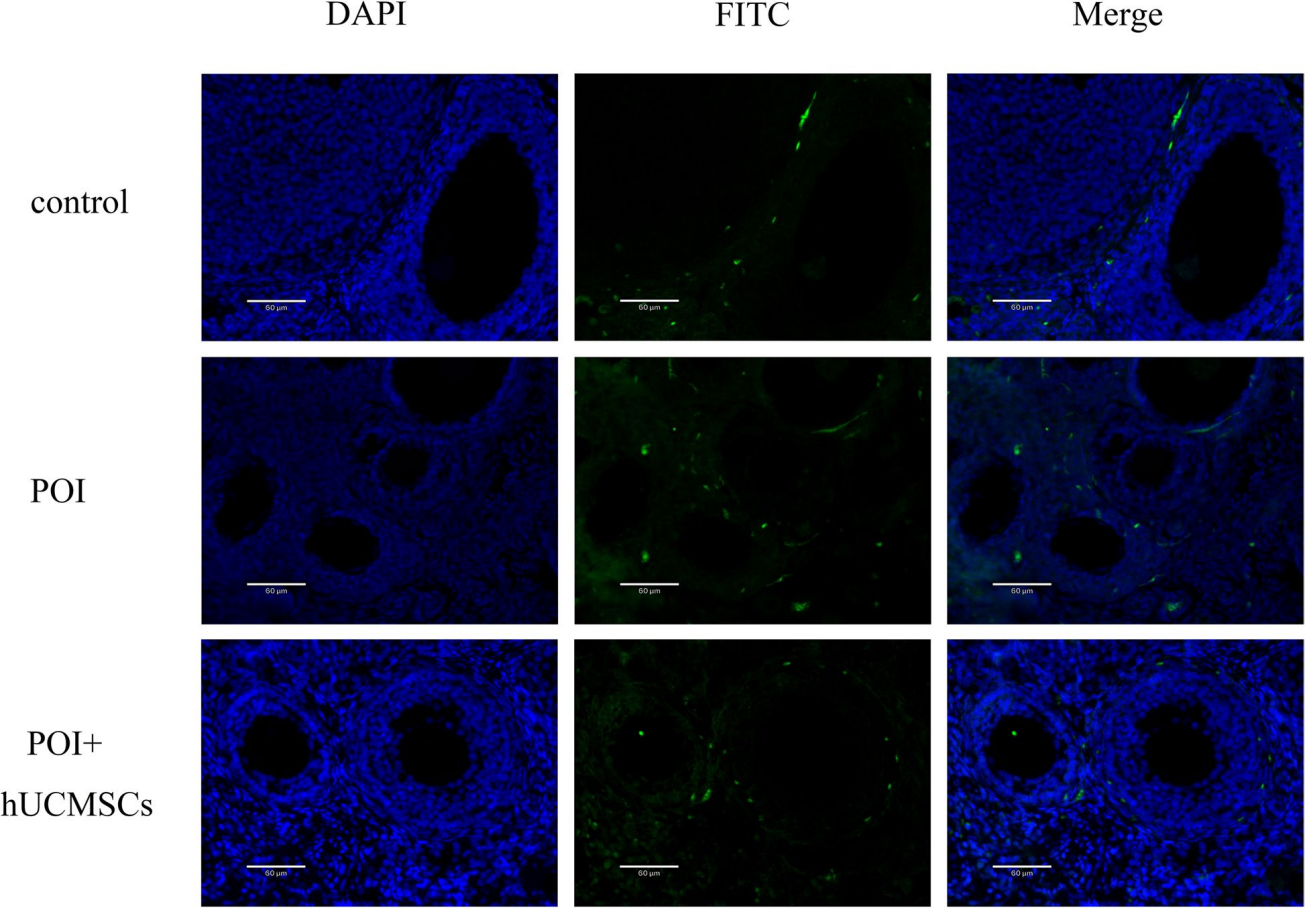
The TUNEL detection kit was used to identify the apoptosis of TICs in the ovaries of all the groups. The FITC staining indicated apoptotic cells. The nucleus was indicated by blue fluorescence while it was stained with DAPI. Magnification × 400. Scale bar: 60 μm. FITC: fluorescein isothiocyanate, DAPI: 4’,6-diamidino-2-phenylindole, POI: premature ovarian insufficiency, hUCMSCs: human umbilical cord mesenchymal stem cells

TUNEL was used to identify the TICs' apoptosis in order to better understand the mechanism underlying their malfunction. According to Fig. 3, more TUNEL-positive cells were found in the ovary of POI rats than in the control group. The hUCMSCs therapy, however, rescues TIC apoptosis.

### Expression of NR4A1 in TICs of POI and hUCMSCs treatment

**Fig. 4:**
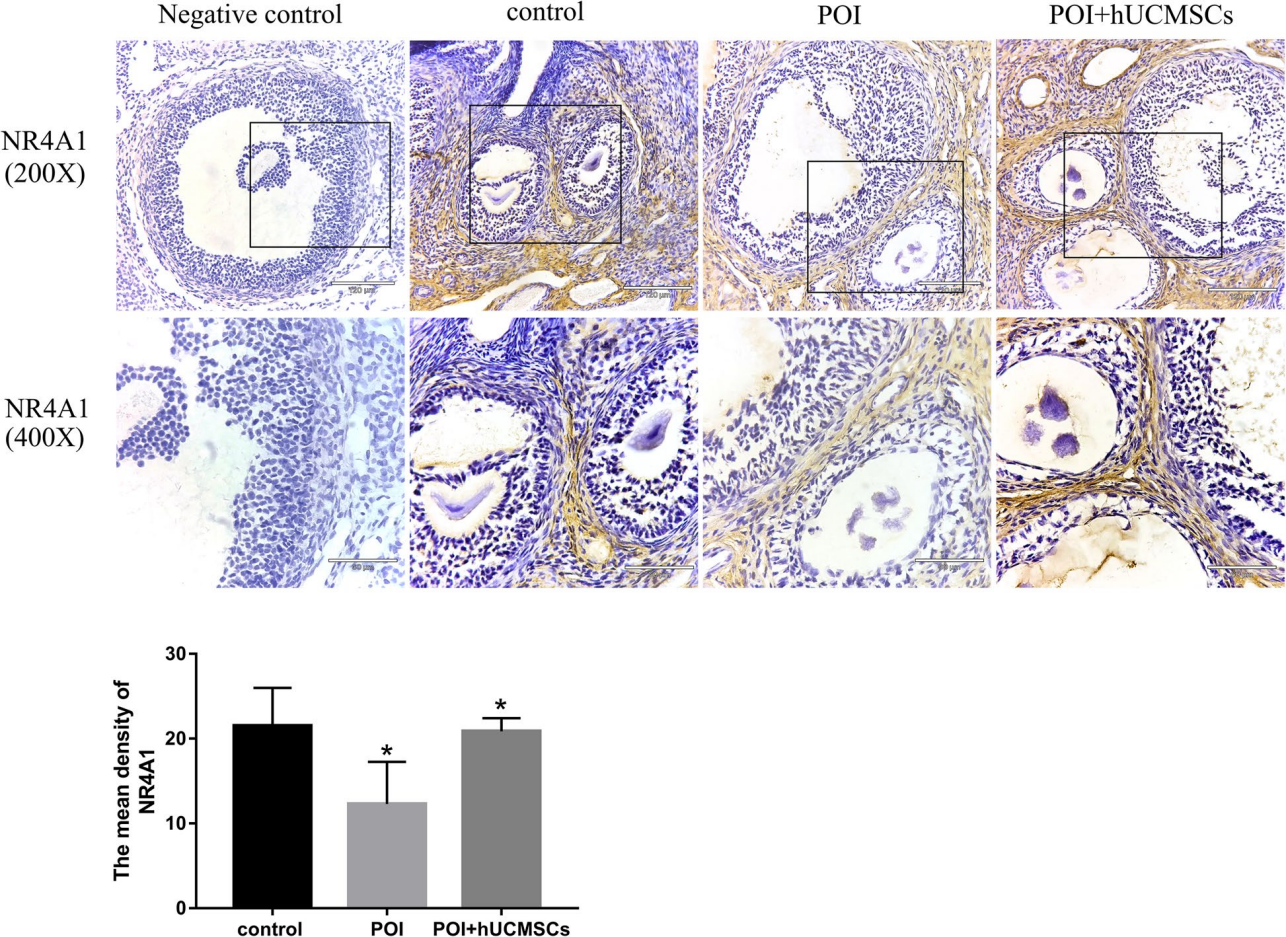
NR4A1 localization in the ovaries as determined by immunohistochemistry. (A)Within the ovary, positive immunoreactive signals of NR4A1 were visualized as brown stains and the nucleus as blue stains (original magnification of 200X and 400X, scale bar = 120 µm and 60 µm). POI: premature ovarian insufficiency, hUCMSCs: human umbilical cord mesenchymal stem cells.*: *P* < 0.05

Numerous studies have demonstrated the role of NR4A1 in other cell types' apoptosis. Our semi-quantitative analysis and immunohistochemistry findings revealed that NR4A1 was mostly expressed in the TICs of ovarian follicles (Fig. 4), but was not seen in granulosa cells. In addition, hUCMSC therapy boosted NR4A1 expression as compared to the ovaries of POI rat. Therefore, NR4A1 might be crucial for the apoptosis of TICs.

### TICs function was ameliorated by hUCMSCs treatment-In vitro model

We isolated TICs and analyzed it in vitro to provide evidence that TICs function is connected to POI. As seen in Supplementary Fig. 2, the TICs morphology of P_1_ generation was classified as having a long spindle shape. ELISA assays showed that E_2_ levels in TICs medium were hardly detectable, but T levels were 6.42 ± 0.19 ng/ml. Cyp17a1 was expressed in the cytoplasm of TICs, but not FSHR, according to the results of immunofluorescent tests.

**Fig. 5:**
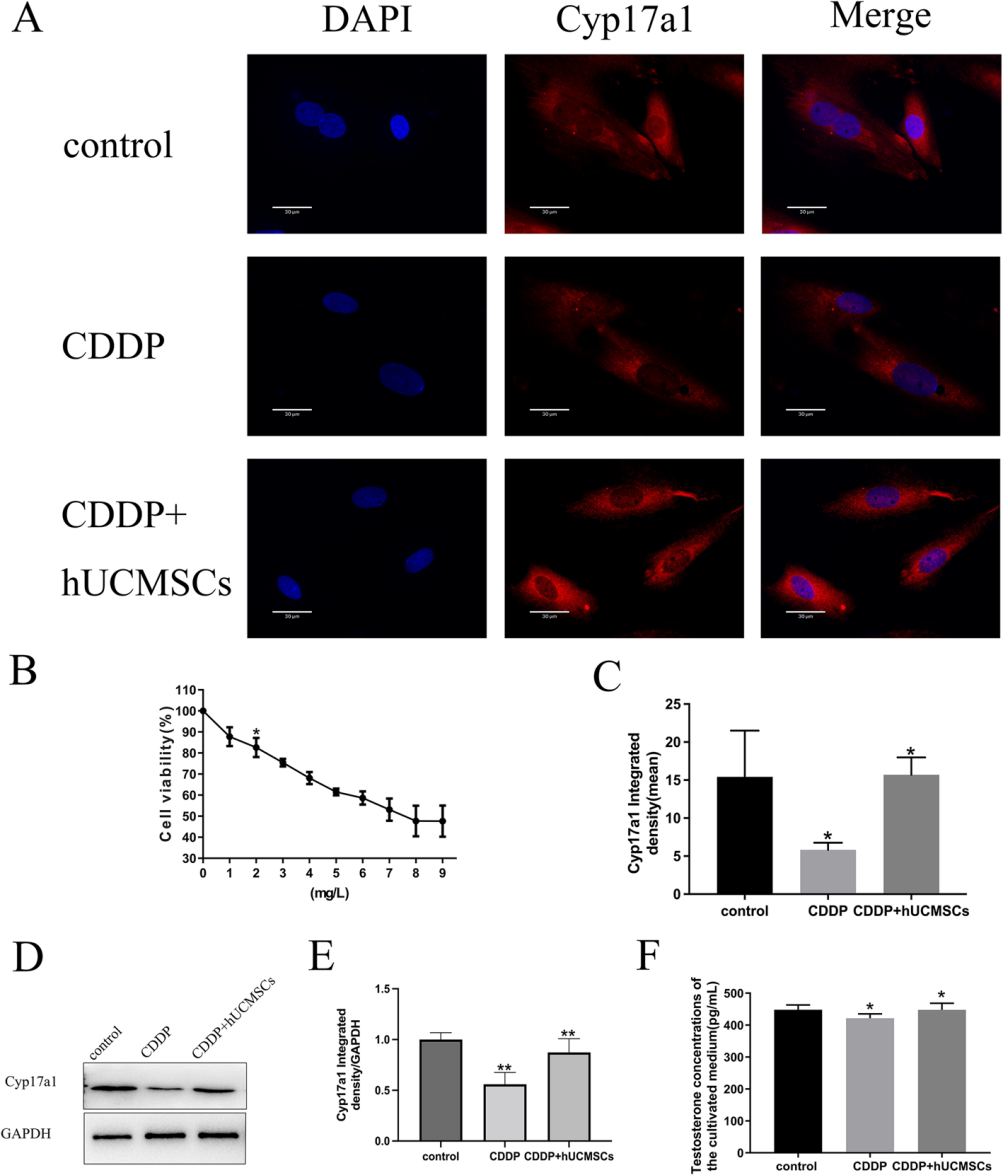
Expressions of Cyp17a1 and the levels of testosterone concentrations in each group were determined and represented as histograms using immunofluorescent assays, western blot, and ELISA. **A**, **C** Nucleus labeled with DAPI appears blue and cells positive for Cyp17a1 were stained with Dylight 549 and appear as red (400X). Scale bar = 30 μm. **B** Survival rates of TICs were detected with the use of the CCK-8 detection kits. Survival rates of TICs (percent) expressed as a function of increasing cisplatin concentrations (mg/L). **D**, **E** Semi-quantitative analysis of Cyp17a1 as measured using western blot. **F** T concentrations within the control, CDDP, and CDDP + hUCMSCs groups as determined using ELISA. CDDP, cisplatin; E_2_, estradiol; T, testosterone; DAPI, 4', 6-diamidino-2-phenylindole; Cyp17a1, Cytochrome P450 Family 17 Subfamily A Member 1. **: *P* < 0.01, *: *P* < 0.05

Responses to 0–10 mg/L cisplatin have been used to evaluate the viability of TICs [29]. We discovered that 2 mg/L concentration of cisplatin causes a mean survival rate of 82.63%. Therefore, this 2 mg/L concentration of cisplatin was used in these in vitro experiments (Fig. 5B).

Results from immunofluorescence and western blot analysis demonstrated that the decreased expressions of Cyp17a1 within the CDDP group were increased after hUCMSCs treatment. Similarly, T concentrations within the cultured medium were decreased by CDDP, while hUCMSCs treatment increased these levels of T (Fig. 5A, C-F). Together, these data provide further support for the role of TICs in POI and its modulation by hUCMSCs.

### Apoptosis of TICs after treatment with cisplatin and hUCMSCs-In vitro model

**Fig. 6:**
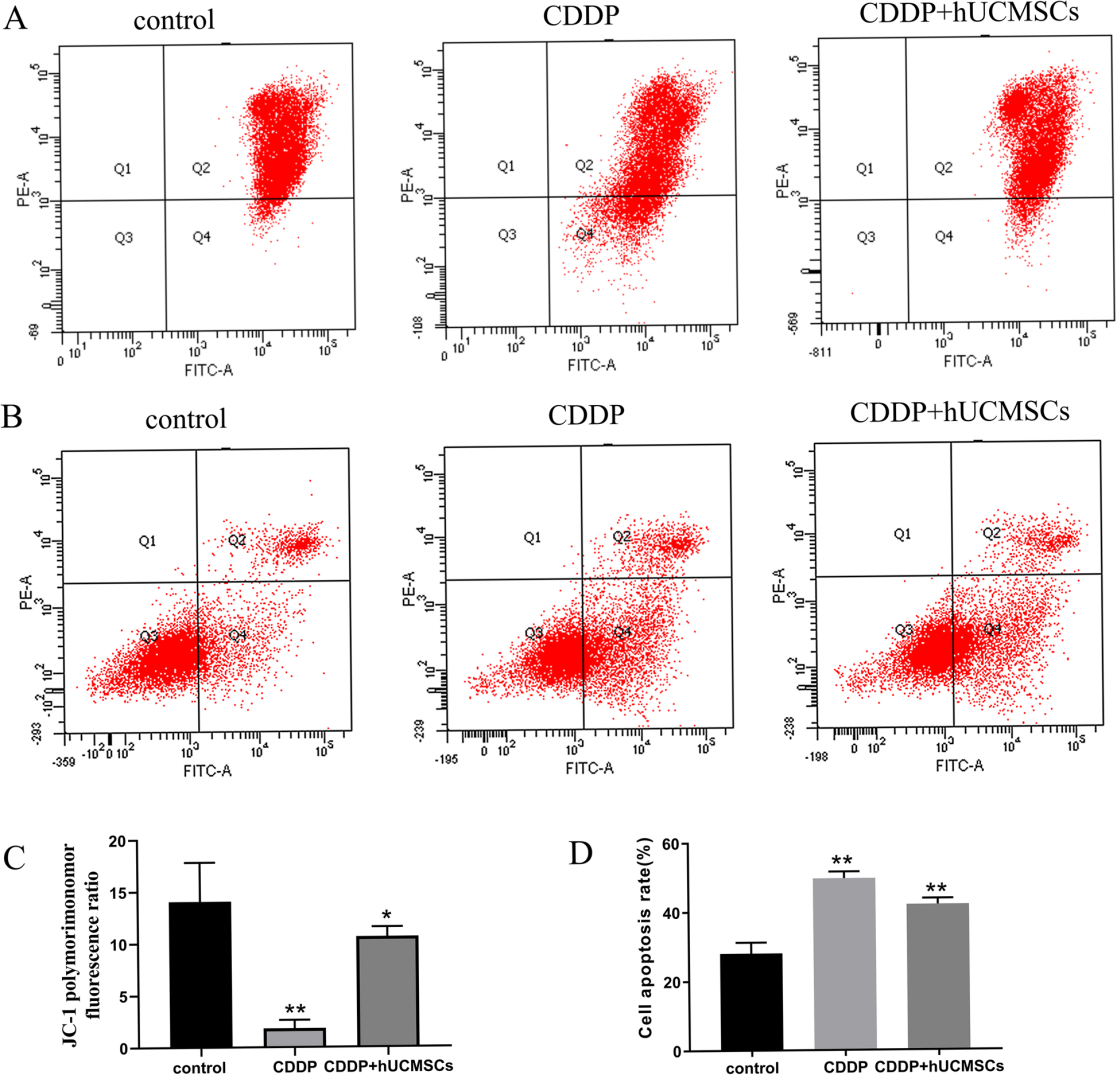
The JC-1 polymorimonomor fluorescent ratios and apoptosis of TICs after cisplatin and hUCMSCs treatment. **A**, **C** Representative pictures of flow cytometry for each group are present. Histograms show JC-1 polymorimonomor fluorescent ratios = Q2/Q4, with Q2 representing the control membrane potential in the form of a polymer and Q4 the green fluorescence of monomers engaged in reduced membrane potentials after the intervention. **B**, **D** Apoptosis of TICs was detected with the use of Annexin V-FITC apoptosis detection kits. Scatter diagrams of PI/Annexin V gating within the different groups are present above. Representative pictures of flow cytometry for each group are present. Statistics analysis of these data is indicated within the histograms. CDDP, cisplatin.**: *P* < 0.01.*: *P* < 0.05

The early stages of apoptosis within cells can be reflected in changes in the mitochondrial membrane potential, which was measured using the mitochondrial membrane potential assay kit with JC-1. Both cisplatin and hUCMSCs had an impact on polymorimonomor fluorescence ratios. Following treatment with hUCMSCs, the ratio of polymorimonomor fluorescence was restored after being lowered by cisplatin (Fig. 6A, C).

Results using the Annexin V-FITC/PI staining kit showed that as compared to the control group, the percent of TICs apoptosis after treatment with cisplatin and hUCMSCs conditional medium were 49.6 ± 2.00% and 42.03 ± 1.72%, respectively (Fig. 6B, D). These results suggest that the apoptosis of TICs resulting from cisplatin was decreased by hUCMSCs treatment.

### The localization and quantitative analysis of NR4A1 among the groups in vitro

**Fig. 7:**
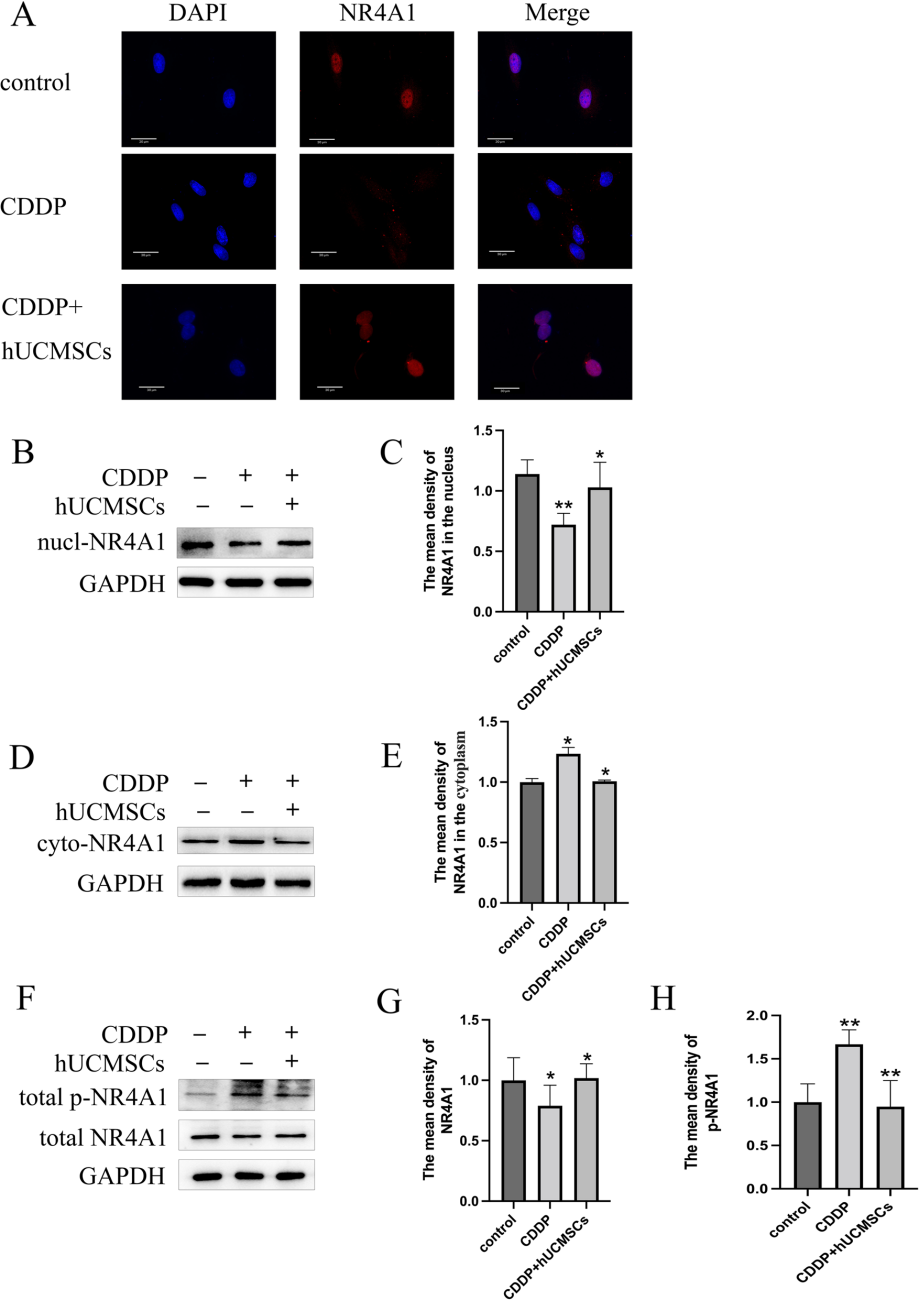
NR4A1 localization in TICs as measured using immunofluorescence. Expression of NR4A1, as well as phospho-NR4A1 levels in each group, were assessed by western blot. **A** Positive immunoreactive NR4A1 signals within the TICs were visualized as red staining and the nucleus as blue staining (original magnification of 400X, scale bar = 30 µm). **B**-**H** The representative bands of western blot are shown in the form of histograms in the co-cultured hUCMSCs and cisplatin-treated TICs. CDDP, cisplatin. *: *P* < 0.05, **: *P* < 0.01

It was observed from immunofluorescent staining that NR4A1 was expressed in the nucleus of these TICs in vitro. Cisplatin and hUCMSCs had an effect on the localization of NR4A1. NR4A1 relocated from the nucleus to the cytoplasm following cisplatin treatment. However, therapy with hUCMSCs can prevent NR4A1 cytoplasmic translocation (Fig. 7A).

We showed NR4A1 expressions in both the nucleus and cytoplasm, as well as phospho-NR4A1 levels in Fig. 7B-H. Treatment with cisplatin resulted in lower total NR4A1 levels but higher phospho-NR4A1 levels. However, these levels exhibited an opposite trend following hUCMSCs treatment. Nucleoplasmic separation was used to separate nuclear proteins from cytoplasmic proteins. The expression of NR4A1 was evaluated by western blot. The findings illustrate that cisplatin increases NR4A1 expression in the cytoplasm while decreasing them in the nucleus.. However, cisplatin-induced NR4A1 cytoplasm translocation can be reversed following hUCMSCs treatment. Therefore, these data were consistent with the immunofluorescent staining.

### The apoptosis of TICs was affected by the antagonist of NR4A1 DIM-C-pPhOH

To further identify some of the specific mechanisms underlying the TICs apoptosis, expressions of Bcl-2, Bax, caspase-9, caspase-3, and Cytc were determined by western blot.

**Fig. 8:**
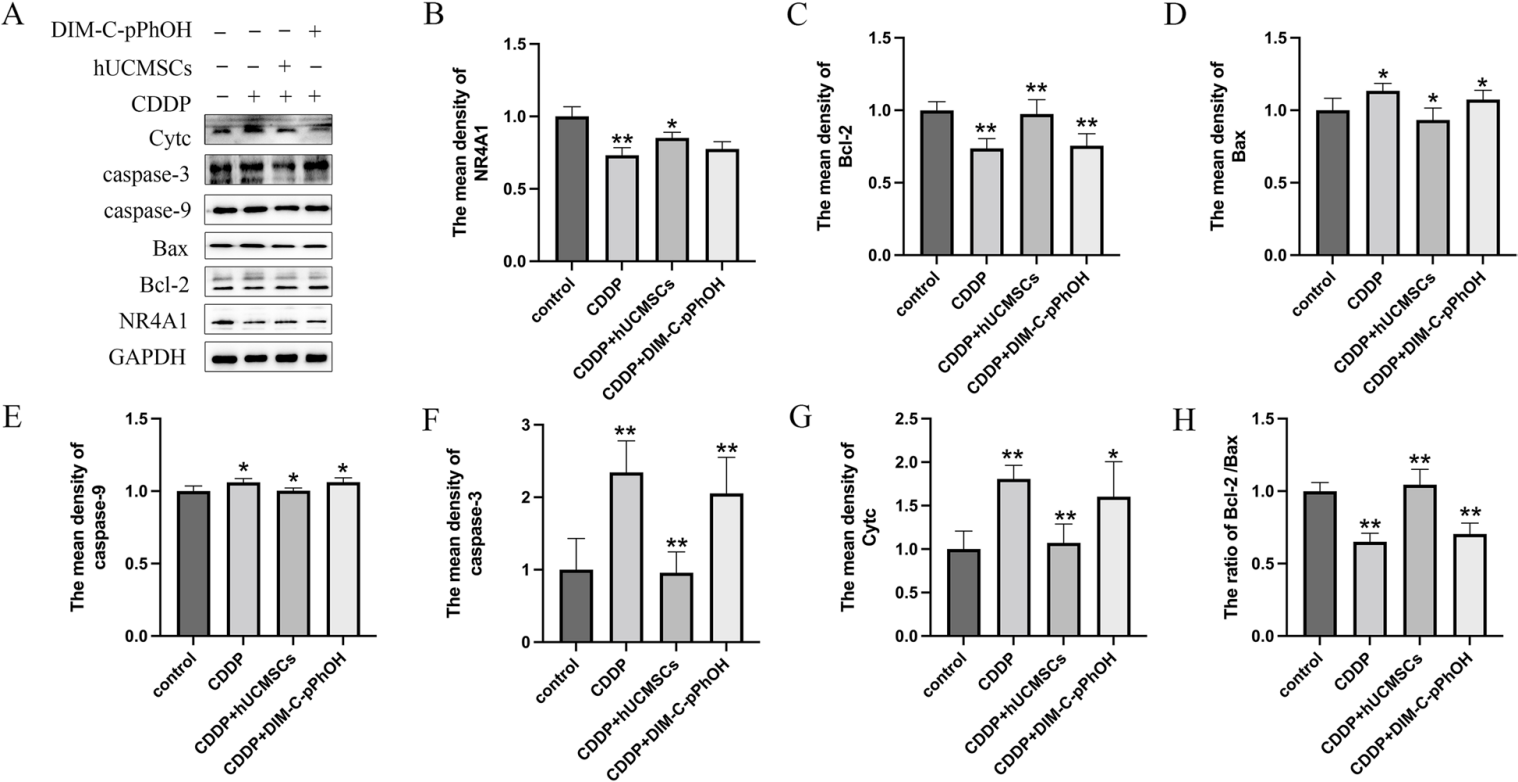
Expression of NR4A1, Bcl-2, Cytc, Bax, caspase-9, caspase-3, and the ratio of Bcl-2/Bax levels are present as the original bands and histograms in TICs following cisplatin treatment, hUCMSCs supernatant co-culture and the antagonist of NR4A1 DIM-C-pPhOH. Statistics analysis of these data is indicated within the histograms. **: *P* < 0.01.*: *P* < 0.05

As shown in Fig. 8, the expression of NR4A1, Bcl-2, and the ratio of Bcl-2 /Bax were dramatically downregulated, whereas the expression of Bax, Cytc, caspase-9, and caspase-3 were significantly increased in the TICs treated by CDDP compared to the control group. We were particularly interested in the upregulation of Bax, Cytc, caspase-9, and caspase-3 following cisplatin treatment as opposed to the downregulation of these protein levels after hUCMSCs treatment, which are recognized markers for mitochondrial mechanisms. Meanwhile, comparatively to the CDDP group, the expression of NR4A1 were increased after hUCMSCs treatment, while the NR4A1 antagonist DIM-C-pPhOH had no effect. Nevertheless, the factors associated with apoptosis, such as Bax, Cytc, caspase-9, and caspase-3, upregulated while downregulated the ratio of Bcl-2/Bax in CDDP + DIM-C-pPhOH treatment group. This result suggested that instead of regulating NR4A1, hUCMSCs could enhance the apoptosis of TICs by raising the ratio of Bcl-2 to Bax.

## Discussion

Increasing evidence has accumulated that hUCMSCs can be used as an effective therapy for POI [30, 31]. To evaluate its therapeutic effect and explore the mechanism, hUCMSCs were isolated from the umbilical cord and their effects on POI were studied. Immunophenotypic characteristics, as well as osteogenesis and lipogenesis tests with these hUCMSCs, were consistent with those published in the literature [32, 33]. In conclusion, hUCMSCs effectively reverse the hormonal levels of E_2_, FSH, LH, and ovarian morphology that were rescued in a cisplatin-induced rat model of POI. HUCMSCs restore the hypoestrogenic and hyper-gonadotropin status caused by POI to normal levels and rescued the ovarian function in POI. Moreover, the results of frozen sections was that hUCMSCs were discovered in the layer of TICs during 72 h, 120 h, and 144 h. According to the results from a previous study, TICs have been demonstrated to be essential in preventing granulosa cell apoptosis [34]. Therefore, it would seem that TICs have a significant impact on POI ovarian dysfunction.

In part, TICs function can be reflected by testosterone (T) levels and expressions of Cyp17a1. This follows, as T is specifically synthesized in TICs and Cyp17a1, is specifically distributed on the endoplasmic reticulum of TICs [35, 36]. In our in vivo experiment, we demonstrated that TICs function was damaged by cisplatin, which were associated with decreased serum T concentrations and Cyp17a1 expression. Increases in T and Cyp17a1 expression following hUCMSCs therapy imply that hUCMSCs restored TICs function. An in vitro model was established by isolating TICs from the ovaries of rats and using expressions of Cyp17a1 and T as a means to assess TICs [25, 37, 38]. With this model, we discovered that both Cyp17a1 expression and T levels in the cultured medium were decreased following cisplatin treatment, while treatment with hUCMSCs raised these levels. These findings were in line with those the in vivo model, and both models offer compelling evidence that TICs dysfunction contributes to POI. HUCMSCs were able to restore the ovarian TICs dysfunction in this POI model.

To identify some of the specific mechanisms of TICs in POI and effects of hUCMSCs treatment, cisplatin was used to induce POI in vivo, then the apoptosis of TICs was detected by TUNEL. After the cisplatin treatment, the apoptosis was elevated in TICs, whereas hUCMSCs treatment decreased the apoptosis. These effects were confirmed following determination of cell apoptosis after cisplatin treatment, along with assessments of hUCMSCs therapy as achieved with the use of Annexin V-FITC/PI kit. Additionally, the follicular atresia, which is assumed to be mediated by apoptosis, was increased in POI rats as showed in Fig. 2F. Accordingly, these results suggest that hUCMSCs decrease TICs apoptosis and restore ovarian function in POI.

To further investigate the mechanisms underlying hUCMSCs effects on the apoptosis of TICs, we next evaluated the function of the orphan nuclear receptor, NR4A1. Numerous studies have been studied on NR4A1, mostly present in the nucleus in normal condition, can transfer to the mitochondria when membrane permeabilization and cell apoptosis [39]. In our study, NR4A1 is expressed in TICs during proestrus of rats according to our immunohistochemical results demonstrating NR4A1 in the ovaries and immunofluorescence results of NR4A1 in the nucleus of TICs. It's interesting to note that it could translocate from the nucleus to the cytoplasm following cisplatin and can be restored by hUCMSCs, as shown by western blot. It proves that TICs’ apoptosis is related to the translocation of NR4A1.

Intriguingly, cisplatin and hUCMSCs had an effect on the expression of phospho -NR4A1. A lot literatures reported that NR4A1 is phosphorylated, which is associated to its translocation [40–42]. We hypothesized that NR4A1 phosphorylation contributed to the TICs’ apoptosis of POI and had an impact on NR4A1 translocation. In fact, in cells undergoing apoptosis, NR4A1 specifically translocated to mitochondrial membranes, as revealed by its punctate staining pattern in the cytoplasm. Therefore, the membrane permeabilization, being assessed by JC-1 after cisplatin treatment, along with assessments of hUCMSCs therapy as achieved with the use of the JC-1 detection kit, was shown in our experiment. Permeabilization of mitochondrial membranes is a critical event that results in release, such molecules include cytochrome c that are critical for apoptosis in many of these pathways [43–47]. The expression of cytc was measured in each group. Cisplatin upregulated the cytc expression and hUCMSCs reduced it in the TICs. It was proposed that cytc released due to the membrane permeabilization is essential for the TICs’ apoptosis during the cisplatin and hUCMSCs treatment.

The cytc elimination was inhibited by Bcl-2, which act antagonistically on Bax and opposing cell apoptosis during the cell apoptosis process [48]. Additionally, it is mainly regulated and activated by the Bcl-2 family, of which pro- and anti-apoptotic molecules (Bax and Bcl-2) are the most common apoptosis markers, and Bax will initiate the apoptosis in the mitochondrial pathway [49]. Cell apoptosis can be detected using the Bcl-2/Bax ratio [50, 51]. To further verify this point, the expression of Bax and Bcl-2 were measured by western blot. The ratio of Bcl-2 /Bax decreased, which facilitated the TICs’ apoptosis. On the contrary, hUCMSCs improved the apoptosis of TICs and elevated the ratio of Bcl-2 /Bax. In addition, the antagonist of NR4A1-DIM-C-pPhOH was treated to TICs in our experiment to further verify the function of NR4A1. DIM-C-pPhOH had no effect on the expression of NR4A1 but induced apoptosis of TICs, following decreased expression of antiapoptotic genes including Bcl-2. However, following DIM-C-pPhOH treatment, pro-apoptotic protein such as cytc, Bax, caspase-9, and caspase-3 increased. In a word, hUCMSCs improved the apoptosis of TICs not by regulating NR4A1 expression but by NR4A1-mediated mitochondrial mechanisms.

## Conclusions

Our results suggest that hUCMSCs can dramatically enhance follicular development and restore the ovarian function, inhibit TICs apoptosis in POI mice by regulating NR4A1 mediated mitochondrial mechanisms. Furthermore, TICs are an important cellular component in follicle development in the ovarian microenvironment. The findings presented in this report offer crucial support for the use of hUCMSCs in the treatment of POI by alleviating the POI ovarian microenvironment.

## Supplementary Information

**Additional file 1: Figure S1.** Identification of human umbilical cord mesenchymal stem cells. **Figure S2.** TICs morphology and the protein levels of Cyp17a1 and testosterone concentrations by western blot and ELISA. **Figure S3.** Morphology and GFP-labeled hUCMSCs were observed in the microscope.

## Data Availability

Data and materials generated and/or analyzed are included in this published article.

## Abbreviations

POI: Premature ovarian insufficiency
TICs: Theca interstitial cells
hUCMSCs: Human umbilical cord mesenchymal stem cells
ELISA: Enzyme-linked immunosorbent assays
HE: Hematoxylin and eosin
TUNEL: Terminal-deoxynucleotidyl transferase mediated dUTP nick end labeling
CDDP: Cisplatin
PBS: Phosphate-buffered saline
FBS: Fetal bovine serum
FSH: Follicle-stimulating hormone
LH: Luteinizing hormone
E_2_: Estradiol; T: testosterone
HLA-DR: Human leukocyte antigen DR
FITC: Fluorescein isothiocyanate
NR4A1: Nuclear receptor subfamily 4, group A, member 1
Cyp17a1: Cytochrome P450 17A1
Bcl-2: B-cell lymphoma-2
Bax: Bcl-2-associated X protein
JC-1: 5,5’,6,6’-Tetrachloro-1,1’,3,3’-tetraethylbenzimidazol-carbocyanine iodide
GFP: Green fluorescent protein
