# Supplementary material for: hUCMSCs reduce theca interstitial cells apoptosis and restore ovarian function in premature ovarian insufficiency rats through regulating NR4A1-mediated mitochondrial mechanisms
Source: Reprod Biol Endocrinol. 2022 Aug 19;20:125. doi: 10.1186/s12958-022-00992-5 (PMC9389823; doi:10.1186/s12958-022-00992-5)
Supplement: Supplementary file 1 — Additional file 1: Figure S1. Identification of human umbilical cord mesenchymal stem cells. Figure S2. TICs morphology and the protein levels of Cyp17a1 and testosterone concentrations by western blot and ELISA. Figure S3. Morphology and GFP-labeled hUCMSCs were observed in the microscope. [file 12958_2022_992_MOESM1_ESM.docx]

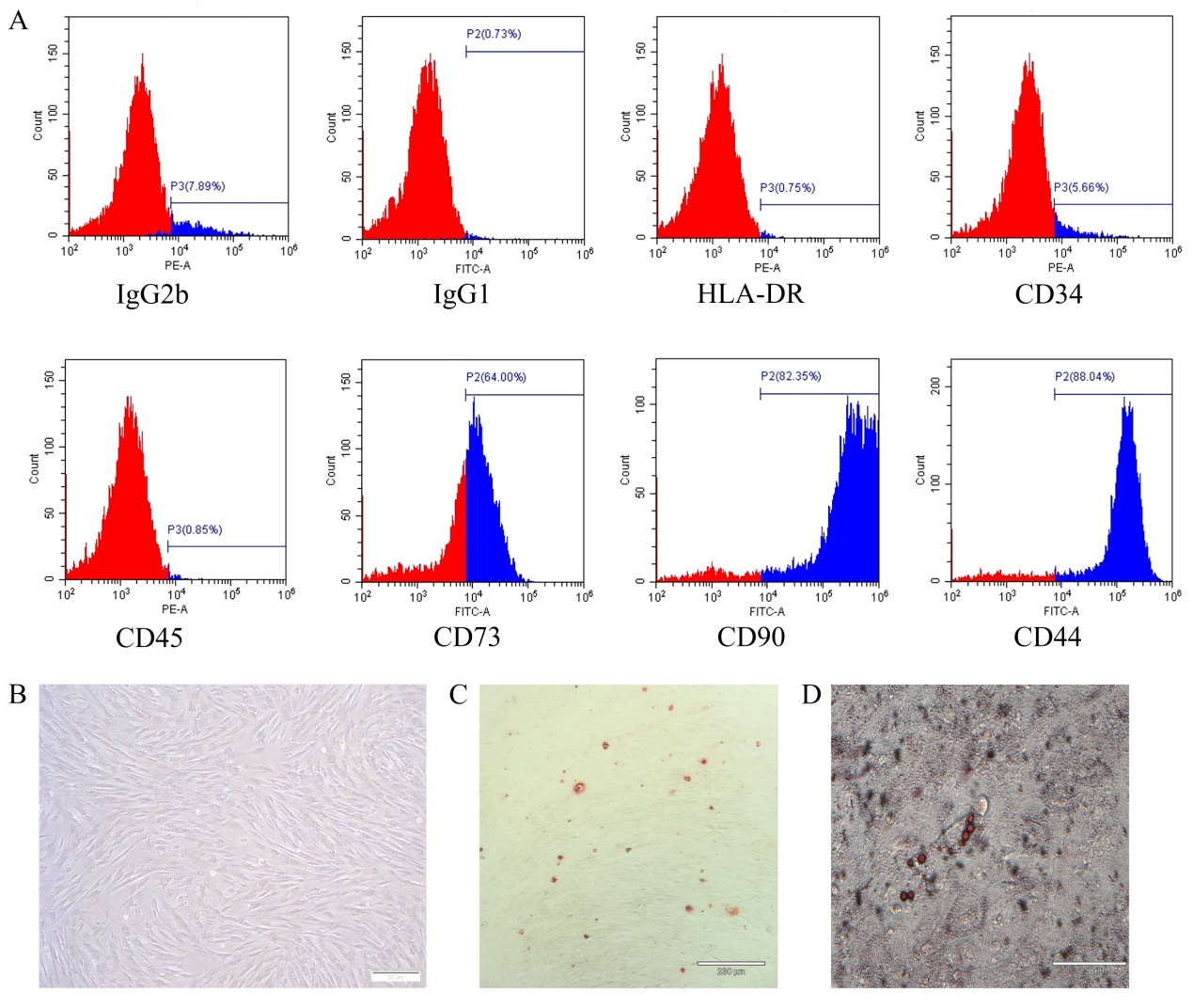


Supplement fig. 1. The characteristics of human umbilical cord mesenchymal stem cells(hUCMSCs). (A) CytoFLEX (Beckman) was used to detect immunophenotypic characteristics of human umbilical cord mesenchymal stem cells (hUCMSCs) lines. Immunophenotypic characteristics of hUCMSCs were determined based on comparisons with the mouse IgG2b kappa isotype (PE) and mouse IgG1 kappa isotype (FITC) controls. Red histograms represent isotype control staining while blue histograms represent the specific expressions of indicated cell surface markers. (B) Morphology of hUCMSCs. Morphological images of hUCMSCs as well as representative images of osteoblastic- and lipogenesis- induced results are shown at 400X magnification. Scale bar=50μm. (C) Osteoblasts differentiated from hUCMSCs were stained with Alizarin red S stains. (D) Adipoblasts differentiated from hUCMSCs were stained with Oil red O stains. Scale bar=230μm. Magnification=100X.


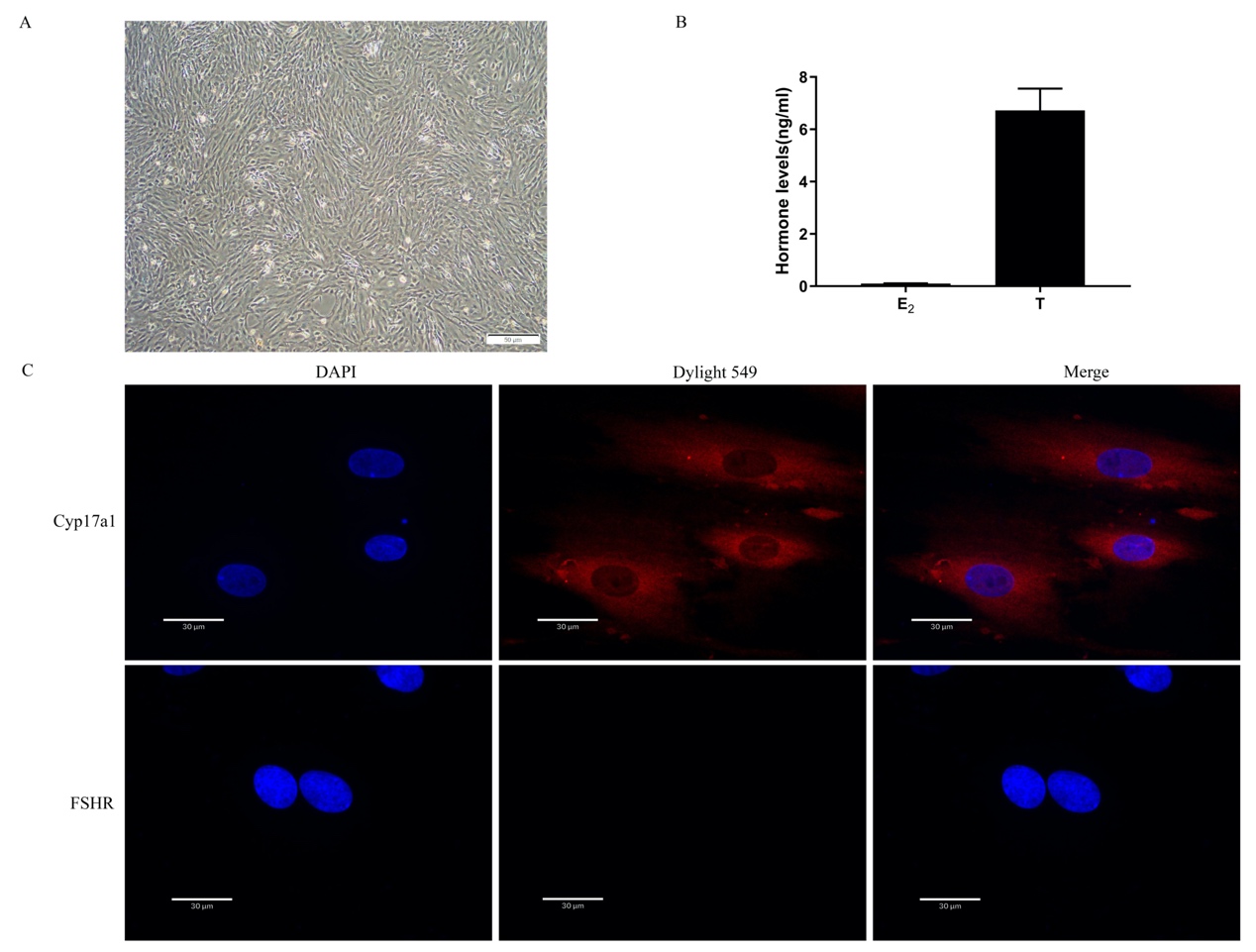


Supplement fig. 2. Morphology and molecular markers of TICs as well as hormone levels used to verify the characteristics of TICs. (A) Morphological observations of TICs are presented at 400X magnification. Scale bar=50μm. (B) Hormonal levels of TICs within the cultured medium were detected with the use of ELISA. (C) Expressions of FSHR, Cyp17a1 were determined with the use of immunofluorescent assays. Nucleus stained with DAPI appear as blue and cells positive for Cyp17a1 and FSHR were stained with Dylight 549 and appear as red (200X). Scale bar=30μm.


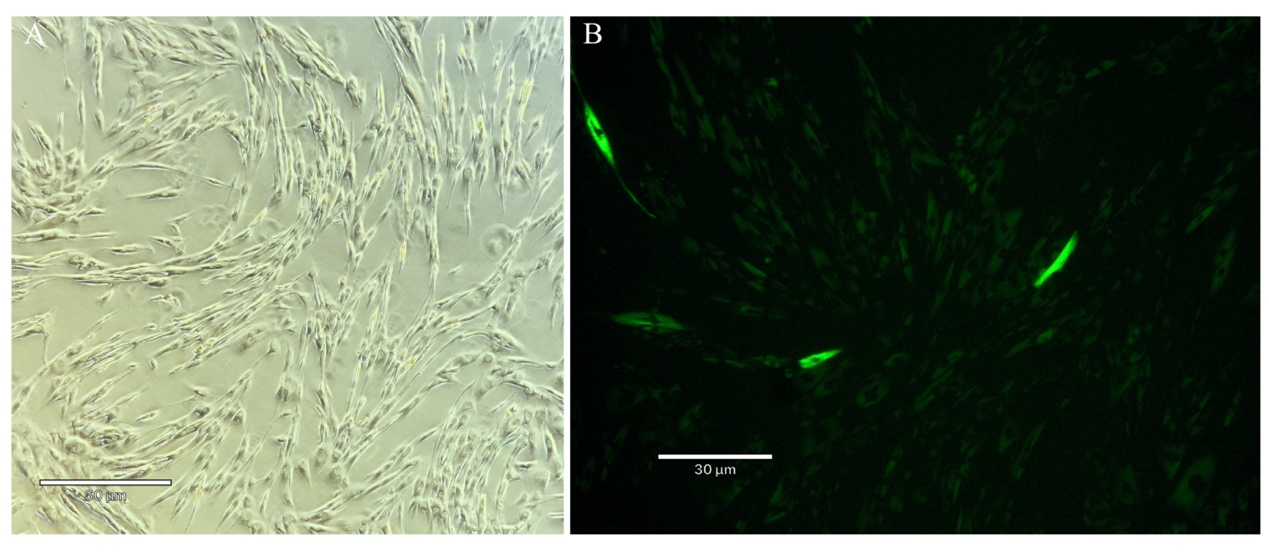


Supplement fig. 3. Morphology and GFP-labeled hUCMSCs were observed in the microscope. (A) Morphological observations of hUCMSCs are presented at 200X magnification. Scale bar=60μm. (B) GFP-labeled hUCMSCs were detected with the lentivirus. Scale bar=30μm.
